# Glutathione Peroxidase and Selenoprotein P Evaluation in Well-Being Assessment After Total Thyroidectomy

**DOI:** 10.3390/ijms26104521

**Published:** 2025-05-09

**Authors:** Emanuela Traini, Francesca Ianni, Edoardo Vergani, Giulia Carnassale, Giuseppe Daloiso, Antonio Mancini, Andrea Silvestrini

**Affiliations:** 1 Ospedale San Carlo di Nancy, GVM Care and Research, 00168 Roma, Italy; etraini@gvmnet.it (E.T.);; 2 Unità Operativa Medicina Interna, Endocrinologia e Diabetologia, Fondazione Policlinico Universitario Agostino Gemelli IRCCS, Università Cattolica del Sacro Cuore, 00168 Roma, Italy; 3 Dipartimento di Scienze Mediche e Chirurgiche, Università Cattolica del Sacro Cuore, 00168 Roma, Italy; 4 Dipartimento di Scienze della Salute e della Vita, Università Europea di Roma, 00163 Roma, Italy

**Keywords:** selenium, hypothyroidism, Selenoprotein P, glutathione peroxidase

## Abstract

It is known that a percentage of patients who undergo total thyroidectomy, approximately 16–34%, complain of symptoms of hypothyroidism or altered quality of life (QoL) despite achieving normal serum TSH values. The present study aimed to identify whether the level of selenium could be responsible for this phenomenon. This pilot cohort study included 44 thyroidectomized outpatients. All patients underwent surgery for benign disease. In this study, no patients with a history of autoimmunity, malignancy, or other conditions that could affect well-being, absorption, or selenium intake were included. Serum levels of TSH, fT3, fT4, Selenoprotein P (SelP), and glutathione peroxidase 3 (GPx3) were measured. The patients also completed the ThyPRO-39 questionnaire to assess their QoL. A strong and significant direct correlation was found between SelP and GPx3 (r = 0.88). However, no correlation was found between hormonal status and SelP or GPx3. Analysis of ThyPRO-39 results did not show clinically significant differences between items nor a correlation with thyroid hormone levels, except for symptoms of hypothyroidism. Interestingly, a significant direct correlation was observed between SelP and tiredness, as well as between GPx3 and tiredness. Furthermore, the fT3/fT4 ratio was correlated with worsening symptoms of hypothyroidism. The results suggest that the selenium status, in turn related to antioxidant activities, as reflected in SelP and GPx3 levels, may be associated with the QoL tiredness domain in thyroidectomized patients, despite normal levels of thyroid hormones. More research is needed to elucidate the role of selenium in the persistent symptoms experienced by some thyroidectomized patients.

## 1. Introduction

The standard replacement therapy for hypothyroidism after total thyroidectomy is synthetic thyroxine (i.e., levothyroxine, L-T4). Although most patients experience good health with appropriately dosed L-T4 therapy, a subset of patients, approximately 16–34%, report persistent symptoms of hypothyroidism or a decreased quality of life compared to their pre-thyroidectomy state. This occurs even though normal serum thyroid stimulating hormone (TSH) concentrations are achieved [1,2].

Thyroxine (T4) is a prohormone with low intrinsic activity, secreted by the thyroid gland and subsequently converted by deiodinases in target tissues into triiodothyronine (T3), the biologically active thyroid hormone. Persistent symptoms of hypothyroidism, despite normal serum TSH levels, may indicate that L-T4 therapy is insufficient to fully restore physiological levels of thyroid hormone within tissues. After total thyroidectomy, the secretion of circulating T3 from the thyroid gland ceases, accounting for approximately 20% of total body T3 production, along with the cessation of other thyroid metabolites. This disruption may lead to hormonal imbalances in predisposed individuals. Furthermore, some studies have explored factors that could predispose individuals to tissue hypothyroidism, even in the presence of normal TSH levels. One such factor is the polymorphism of the enzyme deiodinase D2 (DIO2), which converts T4 into the active form T3 [3]. Selenium, a trace mineral incorporated into approximately 25 different types of selenoproteins in humans, acts as a cofactor for the deiodinases DIO1, DIO2, and DIO3, which are involved in the activation and deactivation of thyroid hormones [4]. Furthermore, it also acts as a cofactor for key antioxidant enzymes such as thioredoxin reductase and glutathione peroxidase (GPx) [5].

Glutathione peroxidase (GPx) is a crucial enzyme that protects cells from oxidative damage by catalyzing the reduction of hydrogen peroxide and organic hydroperoxides by using reduced glutathione (GSH) as a substrate. There are multiple isoforms of GPx, each exhibiting distinct substrate specificities and tissue distributions. Selenium acts as an essential cofactor for most GPx isoforms; therefore, selenium deficiency can alter GPx activity, increasing the risk of diseases related to oxidative stress. In humans, plasma glutathione peroxidase (GPx3), also known as extracellular glutathione peroxidase, is encoded by the *GPX3* gene [6].

When analyzing selenium’s role in thyroid hormone metabolism, Selenoprotein P (SelP) reflects body levels of selenium quite accurately, serving as an indicator of selenium status [5]. SelP is a selenocysteine-containing protein synthesized and secreted primarily by the liver and serves as the primary transporter of selenium in the bloodstream. Its main function is to deliver selenium to various tissues, thus playing a critical role in maintaining selenium homeostasis and ensuring sufficient selenium availability for selenoprotein synthesis in different tissues [7].

Both GPx3 and SelP are selenoproteins, which means that they incorporate selenium in the form of the amino acid selenocysteine. Overall, GPx3 and SelP are key components of the body’s antioxidant defense system, with functions closely related to selenium metabolism and the maintenance of cellular redox balance. Consequently, dysregulation of GPx3 and SelP has been implicated in various pathological conditions related to oxidative stress and selenium deficiency [7].

The present study aims to verify, in a population of patients undergoing total thyroidectomy, whether the availability of selenium is correlated with the following:

- The antioxidant level, analyzing the relationship between SelP and GPx3 (primary endpoint).
- Quality of life (QoL), evaluated using a thyroid-specific QoL questionnaire (secondary endpoint).
- Thyroid hormones (secondary endpoint).

## 2. Results

Table 1 reports the mean ± standard deviation (SD) values of general parameters such as age and thyroid hormones (and their ratio), along with SelP and GPx3.

**Figure 1:**
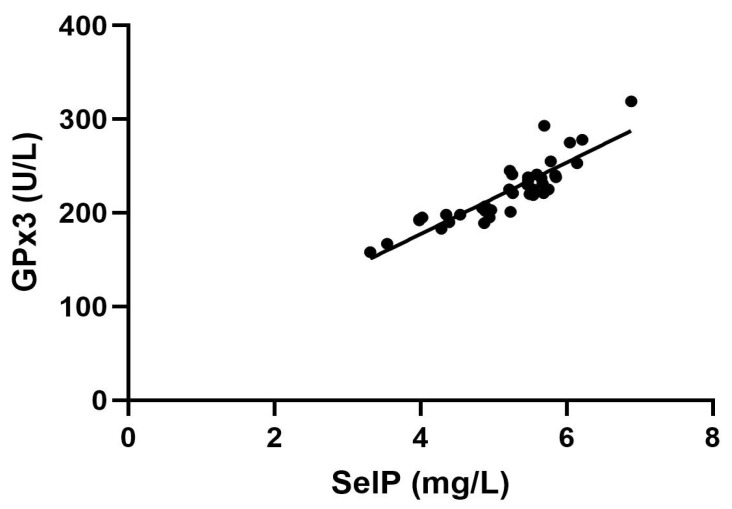
Relationship between glutathione peroxidase activity (GPx3) and Selenoprotein P (SelP) concentrations. A positive correlation analysis is reported between SelP (mg/L) and GPx3 (U/L). *p* < 0.01.

A strong direct correlation (r = 0.88) between SelP and GPx3 was recognized as significant (Figure 1), but no correlation was found between hormonal status, SelP or GPx3, and BMI.

Table 2 shows the results of ThyPRO-39 in our population of thyroidectomized patients.

**Figure 2:**
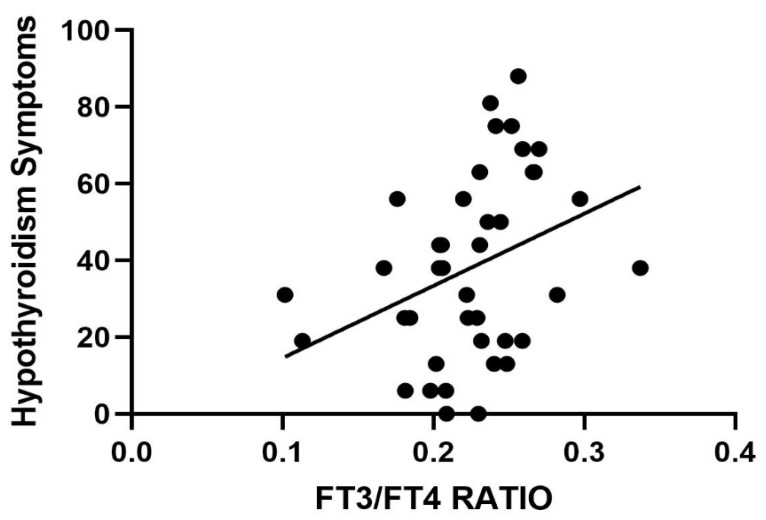
Correlation between the fT3/fT4 ratio and the hypothyroidism symptoms reported in the ThyPRO-39 questionnaire. *p* < 0.01.

Once again, no correlation with thyroid hormones was highlighted. However, when considering the fT3/fT4 ratio and the ThyPRO-39 evaluation, a significant direct correlation emerged between the ratio and the symptoms of hypothyroidism (Figure 2).

**Figure 3:**
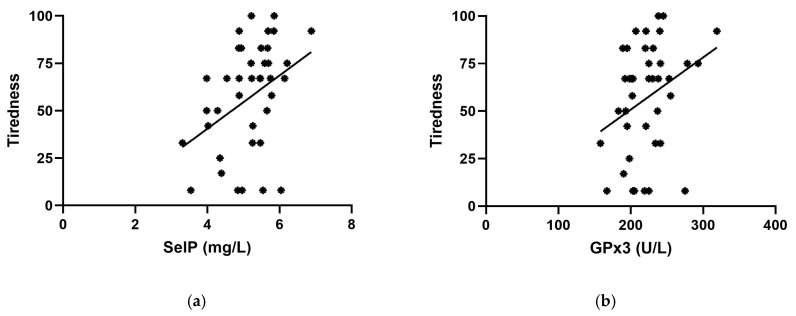
Relationship between tiredness and SelP (**a**) or tiredness and GPx3 (**b**). Parameters affected by tiredness: (**a**) correlation between tiredness and SelP (*p* = 0.01); (**b**) correlation between tiredness and GPx3 (*p* = 0.05). SelP: Selenoprotein P; GPx3: plasma glutathione peroxidase.

When analyzing the correlation between selenium status (i.e., SelP), GPx3, and ThyPRO-39 items, a significant direct correlation was obtained between SelP and tiredness and between GPx3 and tiredness (Figure 3).

## 3. Discussion

The present study offers new insights into the role of selenium status in patients who underwent total thyroidectomy. We measured SelP, which represents the most common selenoprotein that can be measured in plasma and is therefore a reliable indicator of selenium intake [7], and GPx3, as a marker of oxidative stress/antioxidant response. Accordingly, an adequate intake of selenium is necessary for the proper functioning of GPx3. The tissue conversion efficiency of thyroid hormones was evaluated using the fT3/fT4 ratio. The perceived clinical state of hypothyroidism was assessed by administering to cohorts a quality-of-life questionnaire for patients with thyroid disease (ThyPRO-39) [8]. By correlating the results acquired from the evaluation of these parameters in a homogeneous population of patients with a biohumoral state of euthyroidism (TSH level within the normal range), we verified whether selenium deficiency may be responsible for inadequate conversion of fT4 into fT3 at the tissue level and whether there is a relationship with the development of hypothyroidism symptoms perceived by the patient.

Surprisingly enough, the fT3/fT4 ratio was correlated with a worsening of hypothyroidism symptoms on the ThyPRO-39 questionnaire. Furthermore, the tiredness scale was directly correlated with the SelP and GPx3 levels.

It can be hypothesized that, at the tissue level, the conversion of T4 into T3 may differ from the systemic conversion. Therefore, measuring antioxidant parameters downstream of hormonal action, such as GPx3, could provide additional information on thyroid hormone measurement. The ThyPRO-39 questionnaire is a concise form derived from the original ThyPRO, which has been available in several languages since 2009, including Italian [9]. We specifically used this thyroid-related QoL to overcome the intrinsic limitations of general health-related QoL, seeing that QoL is subjective and can prove a challenge to measure [10]. We also narrowed the scales down to ameliorate the analysis in this particular setting of patients thyroidectomized for benign thyroid diseases, excluding eye symptoms, goiter symptoms, and cosmetic complaints.

Higher scores of hypothyroidism symptoms were correlated with higher levels of the fT3/fT4 ratio.

Even if this study had a limited size, its methodology rules out bias due to a moment of depressed mood during the patient’s completion of the questionnaire. Alternatively, we would have expected the involvement of other domains, particularly depression.

To our knowledge, all previous studies focusing on selenium concerned hypothyroidism due to thyroid autoimmunity, which is associated with an increased state of oxidative stress. Several studies explored the effect of selenium supplementation on QoL in hypothyroidism due to thyroid autoimmune disease. A recent randomized, placebo-controlled trial, the CATALYST study, a double-blind, multicenter trial, investigated the effect of 12 months of selenium supplementation on disease-specific QoL (ThyPRO-39 questionnaire) in hypothyroid patients with autoimmune thyroiditis, which showed no benefit [11]. Further studies also showed no effect [12,13,14], while others reported improved well-being with selenium supplementation [15,16,17]. The present research is the first study to intentionally exclude the presence of thyroid autoimmunity and thyroid cancer, which could influence the QoL. We aimed to investigate the role of SelP and GPx3 at the thyroid target tissue level, indirectly inquiring about deiodinase activity. We intentionally selected thyroidectomized patients to avoid the thyroid source of T3, while remaining focused on the peripheral tissue level. No clear association was found between thyroid hormones and ThyPRO-39 domains, except for hypothyroid symptoms, which worsened with higher values of the fT3/fT4 ratio. Previous studies evaluating QoL in hypothyroid patients did not find the same correlation, although autoimmune thyroid disease and thyroid cancer were not excluded [18,19]. It can be postulated that our findings could be the results of an adaptation reaction since the function of deiodinases is preferential over other selenoproteins in the case of selenium deficiency. Winther et al. [20] in their study enrolled 491 euthyroid subjects who were randomized to receive 100 μg (n = 124), 200 μg (n = 122), or 300 μg (n = 119) selenium-enriched yeast or matching yeast-based placebo tablets (n = 126); the authors showed that selenium supplementation, compared to a placebo, has no significant effect on the fT3/fT4 ratio. They concluded that selenium supplementation is not warranted under conditions of marginal selenium deficiency. Our study reveals a significant correlation between SelP and GPx3. The strength of this correlation suggests that SelP levels could serve as a reliable marker of selenium status and, therefore, antioxidant capacity in these patients.

Several experimental and clinical studies have shown that oxidative stress (OS) is related to hyperthyroidism and hypothyroidism [21]. Nevertheless, the mechanisms underlying OS in these two conditions differ: hyperthyroidism is associated with increased ROS production, while hypothyroidism is characterized by reduced availability of antioxidants. Oxidative stress is involved in several complications of hyperthyroidism that affect target tissues [22]. Thyroid hormones themselves can act as oxidants, which can potentially cause DNA damage, a process mitigated by catalase (CAT), likely due to their phenolic structure, which resembles that of steroidal estrogens [23]. Conversely, some thyroid hormone-regulated processes help maintain the oxidative balance through autoloop feedback. Uncoupling proteins UCP-2 and UCP-3 are involved in this regulation, with evidence from plant and animal studies suggesting their antioxidant effects [24]. Thyroid hormones influence the lipid composition of rat tissues, which in turn affects their vulnerability to oxidative stress (OS). However, the response is tissue-specific, and conflicting effects of T3 and T4 have been reported [25,26]. Furthermore, the effects of hyperthyroidism on antioxidant enzyme activity, including Mn-, Cu-, or Zn-superoxide dismutase (SOD), CAT, and GPx, vary by tissue type, with T3 and T4 exerting distinct influences depending on the tissue analyzed [27]. On the other hand, data on hypothyroidism and OS in humans are inconsistent. A study of patients with primary hypothyroidism revealed increased levels of nitric oxide (NO) and elevated plasma levels of malondialdehyde (MDA), which is a marker of OS resulting from lipid peroxidation [28]. Elevated MDA levels were also observed in cases of subclinical hypothyroidism [29]. In this context, the increased OS was mainly attributed not only to reduced antioxidant levels but also to disruptions in lipid metabolism, as a significant correlation was found between MDA levels and LDL-cholesterol, total cholesterol, and triglyceride levels. Total antioxidant status (TAS) was similar across patients with overt hypothyroidism, subclinical hypothyroidism, and healthy controls. Other authors reported the OS in subclinical hypothyroidism, as shown by reduced aryl-esterase and increased TBARS and CAT, but they attributed this pattern to hypercholesterolemia [30]. Lastly, a study involving patients with subclinical hypothyroidism secondary to Hashimoto’s thyroiditis found no difference in baseline MDA levels between hypothyroid patients and healthy controls [31]. It is important to interpret studies involving thyroiditis with attention, as both tissue and systemic inflammation are present in such conditions.

The method used to induce hypothyroidism also influences the findings related to OS (surgical thyroidectomy vs. drug-induced hypothyroidism) [32,33,34]. Other research has focused on oxidative damage in specific organs, particularly the liver, bone, skeletal muscle, and heart. Cardiomyocyte metabolism is dependent on serum fT3, as these cells have minimal deiodinase activity [35]. Hypothyroidism has been associated with varying effects on antioxidant levels in cardiomyocytes, including increased, decreased, or unchanged levels of SOD isoforms, GPx, GSH, and vitamin E [36].

In the present study, our cohort exhibited normal thyroid function; therefore, the positive correlation between fT3 and GPx3 levels could be an early indicator of oxidative unbalance. In such a situation, the activity of GPx3 plays a compensatory mechanism to counteract the initial oxidative stress condition [37]. Since selenium plays a permissive role in the activity of GPx3, it could be speculated that, over time, selenium deficiency or increased radical production could cause a dangerous uncompensated status (i.e., an OS condition).

GPX activity proved to be central even in recent non-thyroidal models, where dysregulation of antioxidant defenses and enhanced ROS production can trigger ferroptosis and tissue injury [38]. It is evident that the oxidative balance is not solely dependent on selenoproteins but rather arises from a more intricate interplay of biochemical and cellular processes. Compelling evidence from animal models suggests that combined supplementation with selenium and other trace elements, such as zinc, may exert synergistic antioxidant effects and resilience against oxidative insults [39]. While our study focused uniquely on selenium-related biomarkers with no data on possible therapies, future works might explore whether multi-nutrient interventions offer superior clinical benefits to the sole selenium. For instance, in a recent study, *Ginkgo biloba* extract was shown to alleviate pesticide-induced testicular injury by modulating SKP2 and Beclin1 signaling pathways to reduce OS-related autophagy [40]. Finally, it is worth remembering that oxidative stress-mediated apoptosis mechanisms highlight the complexity of cellular responses to oxidative insults with possible systemic involvement. Recent single-cell transcriptomic studies have demonstrated that environmental exposure to redox-active metals such as vanadium can significantly alter mitochondrial function, immune responses, and antioxidant defenses both in the liver and the kidney [41,42].

Despite these findings, this study has some limitations: the small number of patients, the heterogeneous age, the absence of a control group of euthyroid non-thyroidectomized patients, and the lack of intracellular markers for thyroid hormone activation. The current results are only observational associations and randomized controlled trials are needed to verify the eventual clinical utility of selenium supplementation in this population. Additionally, reference values for SelP and GPx3 in humans are not well-established. Furthermore, no univocal explanation for OS can be determined, as it is not solely related to thyroid hormone levels, BMI, or other comorbidities. However, it could be indicative of low-grade inflammation, potentially linked to the self-reported sense of not experiencing well-being as captured in the questionnaire. The lack of significant correlations between specific ThyPRO-39 scales and SelP or GPx3 may be attributed to different factors. First, the sample size of our study was rather small, and this may have limited the statistical power to detect subtle associations. Furthermore, ThyPRO-39, although validated, may not be adequately sensitive to evidencing small differences in well-being related specifically to oxidative stress or selenium status. Finally, the scales are designed to detect clinical symptoms related to thyroid functions rather than micronutrient-specific dysfunctions. These points suggest that future studies should be performed in larger cohorts and, when possible, analyze more targeted patient-reported outcome measures.

## 4. Materials and Methods

### 4.1. Study Protocol and Population

The study protocol was submitted and approved by the local ethics committee (prot. n. 205/CE Lazio1). We conducted a pilot cohort study, selecting 44 thyroidectomized outpatients (4 men and 40 women) referred for endocrinological follow-up at the Center for Thyroid Disease of the San Carlo di Nancy (GVM Care & Research) University Hospital in Rome, in the timeframe between 1 January 2023 and 30 April 2024.

All patients were under replacement thyroxine (L-T4) treatment for previous total thyroidectomy because of benign thyroid disease (simple multinodular goiter, toxic multinodular goiter, pre-toxic multinodular goiter).

Patients aged 18–75 years, who were previously (at least 12 months before the inclusion) thyroidectomized, with adequate replacement thyroxine treatment demonstrated by TSH levels ranging from 0.35 to 3 μU/mL, at least in two different blood samples in the last 12 months beforehand, were included in this study. The exclusion criteria were the evidence of thyroid autoimmunity and/or thyroid cancer at the final histology, chronic liver insufficiency and chronic kidney failure (based on blood chemistries and urine analyses), active malignancies, smoking habit, use of supplements providing >50 μg Se/day in the past 6 months, body mass index (BMI) ≥ 40 kg/m^2^, low T3 syndrome due to malnutrition, or other chronic diseases.

All patients approved and signed informed consent, and they were subsequently subjected to a clinical history assessment, blood test, and the ThyPRO-39 questionnaire.

### 4.2. Biochemical and Hormone Parameter Assays

The following data were collected for each patient: sex, age, BMI, medical history, current drug therapy, serum TSH, fT3, fT4, SelP, GPx3, and score on ThyPRO-39 questionnaire items. Free thyroxine (fT4), free triiodothyronine (fT3), and TSH levels were measured by an immunoassay in electrochemiluminescence using an Elecsys System 2010 (Roche Diagnostics, ECLIA, Modular E, Indianapolis, IN, USA); reference ranges were 8.5–15.5 pg/mL for fT4, 2.3–4.2 pg/mL for fT3, and 0.35–2.80 mIU/L for TSH. The functional sensitivity of TSH was 0.03 μU/L (coefficient of variation, CV 20%) and the measuring range was 0.005–100 mUI/l. The functional sensitivity of fT3 was 0.40 pg/mL (20% CV) and the range was 0.40–50. The functional sensitivity of fT4 was 0.92 pg/mL (20% CV) and the measuring range was 0.9–75 pg/mL. Selenoprotein P (SelP) levels were measured in serum by a quantitative method using a human SelP ELISA kit (selenOtest, selenOmed GmbH, Berlin, Germany), with a detection range of 5–700 mg/L and a sensitivity of 10 mg/L. The sample preparation and assay procedures were performed according to the manufacturer’s instructions. GPx3 activity in serum samples was measured using a coupled enzymatic assay that monitors NADPH consumption spectrophotometrically [38]. Briefly, the activity of GPx3 is indirectly determined by the rate of NADPH oxidation at 340 nm, which occurs as glutathione reductase catalyzes the regeneration of consumed glutathione during the GPx3-mediated reduction of H_2_O_2_ [43].

### 4.3. Questionnaire Administration

Each patient received the ThyPRO-39 questionnaire consisting of 39 questions, grouped into 13 multi-item scales [8,9,44]. The ThyPRO scales cover the following aspects: symptoms of goiters, symptoms of hyperthyroidism, symptoms of hypothyroidism, eye symptoms, tiredness, cognitive problems, anxiety, depression, emotional susceptibility, impairment of social life, impairment of daily life, cosmetic complaints, and overall QoL. Each item is rated on a 5-point scale from 0 (no) to 4 (very) and refers to the last 4 weeks. The raw scale scores are derived by adding the response values (0–4) for all the items in a scale. All scales were derived in a linear transformation in the range of 0–100. Increasing scores indicate decreased QoL. The formula for the linear transformation was “Transformed score = (raw sum score/highest possible raw sum score) × 100” [39]. In our study, goiter symptoms were not collected since patients were thyroidectomized; eye symptoms were not included since they were not correlated with thyroidectomy and tissue euthyroidism; cosmetic complaints and overall QoL impact were not evaluated due to the bias offered by the recent surgical intervention. Therefore, 29 items grouped on 10 scales were included in the statistical analysis.

### 4.4. Statistical Analysis

Statistical analysis was carried out using GraphPad Prism (version 10.2.3, GraphPad, Boston, MA, USA). The D’Agostino and Pearson test was performed to preliminarily evaluate all data distributions in the population studied. Continuous variables were expressed as mean ± SD. If a normal distribution of the data was displayed, the results were analyzed by employing Student’s unpaired *t*-test to evaluate the differences between groups and the Pearson coefficient for correlation analysis. On the other hand, if data did not show a normal distribution, the Mann–Whitney test was performed to study the differences between groups and the Spearman coefficient for correlation analysis. The level of significance was set at 0.05 unless otherwise specified.

## 5. Conclusions

Despite the limitations, this pilot study could represent the basis for a larger longitudinal study aimed at enrolling patients who develop an uncompensated status of oxidative stress as well as exploring the potential role of selenium supplementation in managing SelP and GPX3 levels in such conditions. Thus, the SelP evaluation may offer a marker of selenium deficiency. Furthermore, the study of other biomarkers could be useful to better characterize oxidative stress conditions and their correlation with the clinical status of surgical patients.

In conclusion, monitoring replacement therapy should involve a more comprehensive biochemical evaluation, particularly in patients who do not experience an improvement in well-being despite adequate therapy, as assessed solely by T4 and TSH levels, which is common practice in clinical settings.

## Figures and Tables

**Table 1:** Clinical and demographic characteristics of the population studied. SD: standard deviation; BMI: body mass index; SelP: Selenoprotein P; GPx3: plasma glutathione peroxidase; TSH: thyroid-stimulating hormone; fT4: free thyroxine; fT3: free triiodothyronine.

Parameter	Mean ± SD
Age (years)	56.01 ± 9.54
BMI (kg/m^2^)	27.46 ± 4.96
SelP (mg/L)	5.16 ± 0.76
GPx3 (U/L)	222.08 ± 32.92
TSH (μU/mL)	1.32 ± 1.28
fT4 (ng/dL)	1.40 ± 0.22
fT3 (pg/mL)	3.12 ± 0.61
fT3/fT4 ratio	0.22 ± 0.04

**Table 2:** Results of the ThyPRO-39 questionnaire. The table reports the mean percentage and range of the ThyPRO-39 scales for each item.

Item	Mean % and Range
Hypothyroidism symptoms (4 questions)	38 (0–88)
Hyperthyroidism symptoms (4 questions)	29 (0–100)
Tiredness (3 questions)	57 (8–100)
Cognitive (3 questions)	41 (0–100)
Anxiety (3 questions)	39 (0–100)
Depression (3 questions)	36 (0–100)
Emotional susceptibility (2 questions)	60 (0–100)
Impaired social life (3 questions)	15 (0–83)
Impaired daily life (3 questions)	27 (0–83)

## Data Availability

The data presented in this study are available only on request from the corresponding authors due to the European Union General Data Protection Regulation (GDPR), to ensure the data privacy of the subjects participating in this clinical research project.

## Abbreviations

The following abbreviations are used in this manuscript:

CAT	catalase
fT4	free thyroxine
fT3	free triiodothyronine
GPx3	glutathione peroxidase 3
L-T4	levothyroxine
OS	oxidative stress
QoL	quality of life
SelP	selenoprotein P
SOD	superoxide dismutase
TSH	thyroid stimulating hormone

## References

[1] Kim B.W., Bianco A.C. (2009). For some, L-thyroxine replacement might not be enough: A genetic rationale. J. Clin. Endocrinol. Metab..

[2] Peterson S.J., Cappola A.R., Castro M.R., Dayan C.M., Farwell A.P., Hennessey J.V., Kopp P.A., Ross D.S., Samuels M.H., Sawka A.M. (2018). An Online Survey of Hypothyroid Patients Demonstrates Prominent Dissatisfaction. Thyroid.

[3] Castagna M.G., Dentice M., Cantara S., Ambrosio R., Maino F., Porcelli T., Marzocchi C., Garbi C., Pacini F., Salvatore D. (2017). DIO2 Thr92Ala Reduces Deiodinase-2 Activity and Serum-T3 Levels in Thyroid-Deficient Patients. J. Clin. Endocrinol. Metab..

[4] Balázs C., Rácz K. (2013). The role of selenium in endocrine system diseases. Orvosi Hetilap.

[5] Silvestrini A., Mordente A., Martino G., Bruno C., Vergani E., Meucci E., Mancini A. (2020). The Role of Selenium in Oxidative Stress and in Nonthyroidal Illness Syndrome (NTIS): An Overview. Curr. Med. Chem..

[6] Nirgude S., Choudhary B. (2021). Insights into the role of GPX3, a highly efficient plasma antioxidant, in cancer. Biochem. Pharmacol..

[7] Schomburg L. (2022). Selenoprotein P—Selenium transport protein, enzyme and biomarker of selenium status. Free Radic. Biol. Med..

[8] Watt T., Bjorner J.B., Groenvold M., Cramon P., Winther K.H., Hegedüs L., Bonnema S.J., Rasmussen Å.K., Ware J.E., Feldt-Rasmussen U. (2015). Development of a Short Version of the Thyroid-Related Patient-Reported Outcome ThyPRO. Thyroid.

[9] Watt T., Bjorner J.B., Groenvold M., Rasmussen A.K., Bonnema S.J., Hegedus L., Feldt-Rasmussen U. (2009). Establishing construct validity for the thyroid-specific patient-reported outcome measure (ThyPRO): An initial examination. Qual. Life Res..

[10] Bottomley A. (2002). The Cancer Patient and Quality of Life. Oncologist.

[11] Larsen C., Winther K.H., Cramon P.K., Rasmussen Å.K., Feldt-Rasmusssen U., Knudsen N.J., Bjorner J.B., Schomburg L., Demircan K., Chillon T.S. (2024). Selenium supplementation and placebo are equally effective in improving quality of life in patients with hypothyroidism. Eur. Thyroid J..

[12] Pilli T., Cantara S., Schomburg L., Cenci V., Cardinale S., Heid E.C., Kühn E.C., Cevenini G., Sestini F., Fioravanti C. (2015). IFNγ-Inducible Chemokines Decrease upon Selenomethionine Supplementation in Women with Euthyroid Autoimmune Thyroiditis: Comparison between Two Doses of Selenomethionine (80 or 160 μg) versus Placebo. Eur. Thyroid J..

[13] Eskes S.A., Endert E., Fliers E., Birnie E., Hollenbach B., Schomburg L., Köhrle J., Wiersinga W.M. (2014). Selenite supplementation in euthyroid subjects with thyroid peroxidase antibodies. Clin. Endocrinol..

[14] Karanikas G., Schuetz M., Kontur S., Duan H., Kommata S., Schoen R., Antoni A., Kletter K., Dudczak R., Willheim M. (2008). No immunological benefit of selenium in consecutive patients with autoimmune thyroiditis. Thyroid.

[15] Gartner R., Gasnier B.C., Dietrich J.W., Krebs B., Angstwurm M.W. (2002). Selenium supplementation in patients with autoimmune thyroiditis decreases thyroid peroxidase antibodies concentrations. J. Clin. Endocrinol. Met..

[16] Souza L.S.L., Campos R.O., Braga J.S., Filho J.D.S., Ramos H.E., Anunciação S.M., Cassemiro J.F., Rende P.R.F., Hecht F. (2025). Selenium nutritional status and thyroid dysfunction. Arch Endocrinol Metab..

[17] Nordio M., Basciani S. (2017). Myo-inositol plus selenium supplementation restores euthyroid state in Hashimoto’s patients with subclinical hypothyroidism. Eur. Rev. Med. Pharmacol. Sci..

[18] Morón-Díaz M., Saavedra P., Alberiche-Ruano M.P., Rodríguez-Pérez C.A., López-Plasencia Y., Marrero-Arencibia D., González-Lleó A.M., Boronat M. (2021). Correlation between TSH levels and quality of life among subjects with well-controlled primary hypothyroidism. Endocrine.

[19] Larsen C.B., Winther K.H., Cramon P.K., Rasmussen Å.K., Feldt-Rasmussen U., Groenvold M., Bjorner J.B., Hegedüs L., Watt T., Bonnema S.J. (2023). Severity of hypothyroidism is inversely associated with impaired quality of life in patients referred to an endocrine clinic. Thyroid Res..

[20] Winther K.H., Bonnema S.J., Cold F., Debrabant B., Nybo M., Cold S., Hegedüs L. (2015). Does selenium supplementation affect thyroid function? Results from a randomized, controlled, double-blinded trial in a Danish population. Eur. J. Endocrinol..

[21] Resch U., Helsel G., Tatzber F., Sinzinger H. (2002). Antioxidant status in thyroid dysfunction. Clin. Chem. Lab. Med..

[22] Asayama K., Kato K. (1990). Oxidative muscular injury and its relevance to hyperthyroidism. Free Radic. Biol. Med..

[23] Venditti P., Di Meo S. (2006). Thyroid hormone-induced oxidative stress. Cell Mol. Life. Sci..

[24] Hoang T., Kuljanin M., Smith M.D., Jelokhani-Niaraki M. (2015). A biophysical study on molecular physiology of the uncoupling proteins of the central nervous system. Biosci. Rep..

[25] Venditti P., Daniele M.C., Masullo P., Di Meo S. (1999). Antioxidant-sensitive triiodothyronine effects on characteristics of rat liver mitochondrial population. Cell Physiol. Biochem..

[26] Huh K., Kwon T.H., Kim J.S., Park J.M. (1998). Role of the hepatic xanthine oxidase in thyroid dysfunction: Effect of thyroid hormones in oxidative stress in rat liver. Arch. Pharm. Res..

[27] Choudhury S., Chainy G.B.N., Mishro M.M. (2003). Experimentally induced hypo- and hyper-thyroidism influence on the antioxidant defense system in adult rat testis. Andrologia.

[28] Baskol G., Atmaca H., Tanriverdi F., Baskol M., Kocer D., Bayram F. (2007). Oxidative stress and enzymatic antioxidant status in patients with hypothyroidism before and after treatment. Exp. Clin. Endocrinol. Diabetes.

[29] Torun A.N., Kulaksizoglu S., Kulaksizoglu M., Pamuk B.O., Isbilen E., Tutuncu N.B. (2009). Serum total antioxidant status and lipid peroxidation marker malondialdehyde levels in overt and subclinical hypothyroidism. Clin. Endocrinol..

[30] Santi A., Duarte M.M., de Menezes C.C., Loro V.L. (2012). Association of lipids with oxidative stress biomarkers in subclinical hypothyroidism. Int. J. Endocrinol..

[31] Öztürk Ü., Vural P., Özderya A., Karadağ B., Doğru-Abbasoğlu S., Uysal M. (2012). Oxidative stress parameters in serum and low-density lipoproteins of Hashimoto’s thyroiditis patients with subclinical and overt hypothyroidism. Int. Immunopharmacol..

[32] Estévez-Carmona M.M., Meléndez-Camargo E., Ortiz-Butron R., Pineda-Reynoso M., Franco-Colin M., Cano-Europa E. (2013). Hypothyroidism maintained reactive oxygen species-steady state in the kidney of rats intoxicated with ethylene glycol: Effect related to an increase in the glutathione that maintains the redox environment. Toxicol. Ind. Health.

[33] Cano-Europa E., Pérez-Severiano F., Vergara P., Ortiz-Butrón R., Ríos C., Segovia J., Pacheco-Rosado J. (2008). Hypothyroidism induces selective oxidative stress in amygdala and hippocampus of rat. Metabol. Brain. Dis..

[34] Ortiz-Butron R., Blas-Valdivia V., Franco-Colin M., Pineda-Reynoso M., Cano-Europa E. (2011). An increase of oxidative stress markers and the alteration of the antioxidant enzymatic system are associated with spleen damage caused by methimazole-induced hypothyroidism. Drug Chem. Toxicol..

[35] Klein I., Danzi S. (2007). Thyroid disease and the heart. Circulation.

[36] Elnakish M.T., Ahmed A.A.E., Mohler P.J., Janssen P.M. (2015). Role of oxidative stress in thyroid hormone-induced cardiomyocyte hypertrophy and associated cardiac dysfunction: An undisclosed story. Oxid. Med. Cell. Longev..

[37] Silvestrini A., Mancini A. (2024). The Double-Edged Sword of Total Antioxidant Capacity: Clinical Significance and Personal Experience. Antioxidants.

[38] Peng J., Dai X., Zhang T., Hu G., Cao H., Guo X., Fan H., Chen J., Tang W., Yang F. (2024). Copper as the driver of the lncRNA-TCONS-6251/miR-novel-100/TC2N axis: Unraveling ferroptosis in duck kidney. Int. J. Biol. Macromol..

[39] Fedala A., Adjroud O., Abid-Essefi S., Timoumi R. (2021). Protective effects of selenium and zinc against potassium dichromate–induced thyroid disruption, oxidative stress, and DNA damage in pregnant Wistar rats. Environ. Sci. Pollut. Res..

[40] Wang H., Yang F., Ye J., Dai X., Liao H., Xing C., Jiang Z., Peng C., Gao F., Cao H. (2024). Ginkgo biloba extract alleviates deltamethrin-induced testicular injury by upregulating SKP2 and inhibiting Beclin1-independent autophagy. Phytomedicine.

[41] Chen J., Dai X., Xing C., Zhang Y., Cao H., Hu G., Guo X., Gao X., Liu P., Yang F. (2024). Cooperative application of transcriptomics and ceRNA hypothesis: lncRNA-00742/miR-116 targets CD74 to mediate vanadium-induced mitochondrial apoptosis in duck liver. J. Hazard. Mater..

[42] Qiao N., Dai X., Chen J., Cao H., Hu G., Guo X., Liu P., Xing C., Yang F. (2024). Single nucleus RNA sequencing reveals cellular and molecular responses to vanadium exposure in duck kidneys. J. Hazard. Mater..

[43] Flohé L., Günzler W.A. (1984). Assays of glutathione peroxidase. Methods Enzymol..

[44] Zahan A.E., Watt T., Pascanu I., Rasmussen A.K., Hegedüs L., Bonnema S.J., Feldt-Rasmussen U., Bjorner J.B., Nadasan V., Boila A. (2018). The Romanian version of the thyroid-related patient-reported outcomes THYPRO and THYPRO-39. Translation and assessment of reliability and cross-cultural validity. Acta Endocrinol..

